# An audit of diagnosis and treatment of tuberculosis in Ethiopia

**DOI:** 10.4102/phcfm.v6i1.582

**Published:** 2014-08-13

**Authors:** Valerie J. Ehlers, Getahun S. Aragaw

**Affiliations:** 1 Department of Health Studies, University of South Africa, South Africa

## Abstract

**Background:**

Despite the existence of national tuberculosis guidelines (NTG) in Ethiopia, the incidence and prevalence of tuberculosis did not decline markedly. Audits could attempt to determine whether or not healthcare professionals actually implemented these guidelines, as non-implementation could contribute to suboptimal tuberculosis treatment outcomes.

**Aim:**

To evaluate healthcare providers’ implementation of Ethiopia's NTG during the diagnosis and treatment of tuberculosis in order to enhance tuberculosis treatment outcomes.

**Methods:**

A descriptive, cross-sectional study design was used.

**Results:**

Healthcare providers implemented the NTG during tuberculosis diagnosis for female (60.9%; *n* = 67) and male (56.1%; *n* = 69) patients. The correct numbers of anti-tuberculosis pills, complying with the NTG recommendations, were prescribed for 91.8% (*n* = 101) of the women and for 90.2% (*n* = 111) of the men. However, both over- and under-prescriptions of anti-tuberculosis drugs occurred. There was an over-diagnosis of smear-negative pulmonary tuberculosis. Only 2.6% (*n* = 2) of the 76 smear-negative pulmonary tuberculosis patients had been diagnosed correctly.

**Conclusion:**

Implementation of the NTG should be enhanced, especially with regard to the diagnosis of smear-negative pulmonary tuberculosis patients and the correct prescription of anti-tuberculosis drugs. This would help to increase the number of correctly-diagnosed and -treated tuberculosis patients, improve tuberculosis treatment outcomes, decrease the spread of tuberculosis and prevent the development of multi-drug-resistant tuberculosis strains.

## Abstrait

**Application par les prestataires de soins des Directives nationales de l'Ethiopie pour la lutte contre la tuberculose**.

**Contexte:**

Malgré l'existence de Directives nationales contre la tuberculose (DNT) en Ethiopie, l'incidence et la prévalence de la tuberculose n'ont pas beaucoup diminué. On pourrait déterminer par des contrôles si oui ou non les professionnels de la santé ont appliqué ces directives, car la non-exécution de celles-ci pourrait avoir un effet sur les résultats sous-optimaux du traitement de la tuberculose.

**Objectif:**

Evaluer l'application des DNT de l'Ethiopie par les prestataires de soins au cours du diagnostique et du traitement de la tuberculose afin de rehausser les résultats du traitement de celle-ci.

**Méthodes:**

On a utilisé une conception d’étude transversale et descriptive.

**Résultats:**

Les prestataires de soins ont appliqué les DNT au cours du diagnostique de la tuberculose chez les femmes (60.9%; *n* = 67) et chez les hommes (56.1%; *n* = 69). On a prescrit la quantité correcte de comprimés contre la tuberculose, conformément aux recommandations des DNT, à 91.8% (*n* = 101) des femmes et 90.2% (*n* = 111) des hommes. Cependant, il y a eu des cas de prescriptions excessives et insuffisantes de médicaments antituberculeux. Il y a eu un diagnostique excessif de tuberculose pulmonaire à frottis négatif. Seuls 2.6% (*n* = 2) des 76 patients avec une tuberculose pulmonaire à frottis négatif avaient été diagnostiqués correctement.

**Conclusion:**

L'application des DNT devra être augmentée, en particulier en ce qui concerne le diagnostique des patients avec une tuberculose pulmonaire à frottis négatif et la prescription correcte des médicaments antituberculeux. Cela permettrait d'augmenter le nombre de patients tuberculeux bien diagnostiqués et bien traités, d'améliorer les résultats du traitement de la tuberculose, de diminuer la progression de la tuberculose et d’éviter le développement des souches de tuberculose multi-résistante aux médicaments.

## Introduction

Tuberculosis (TB) poses major public health challenges and its incidence continues to increase in many parts of the world, especially in countries in sub-Saharan Africa (SSA). Based on estimates by the World Health Organization^1^ (WHO), 9.27 million new TB cases (139 per 100 000 population) were diagnosed globally during 2007. It was estimated that 44% (4.1 million) of these cases (61 per 100 000 population) were smear positive. Asia accounted for 55% of the global TB cases during 2007,^1^ whilst Africa accounted for 31%.

In low-income countries with high rates of human immunodeficiency virus (HIV) infection, TB prevalence remains high. Although most countries have implemented the internationally-recommended directly-observed treatment short course strategy (DOTS), SSA continued to report high TB incidence and prevalence rates. Thirteen of the 15 countries with the highest estimated global TB incidence are in Africa.^1^ Limited healthcare seeking behaviours on the part of the patients and shortages of healthcare professionals contribute to the TB prevalence rates in Africa.^2^

According to the 2008 WHO estimate,^1^ Ethiopia ranks seventh amongst the countries with a high TB burden. According to Ethiopia's Ministry of Health,^3^ TB is the major cause of morbidity in Ethiopia and the second-highest cause of mortality, superseded only by malaria. The incidence of all types of TB in Ethiopia totaled 379 diagnoses per 100 000 population in 2008, but only 40 794 smear-positive PTB cases were detected.^1^ The highest case detection rate (76.7%) in Ethiopia was documented in the Gambella region and the lowest (20.3%) in the Somali region, whilst it was 68.3% in Addis Ababa, Ethiopia's capital city, during 2008. Mengiste et al.^4^ also reported ineffective diagnosis and treatment of TB in the Tigray region of northern Ethiopia.

Understanding on the part of healthcare workers about the need for three sputum analyses for the diagnosis of TB, as well as its public health significance, is affected by their educational background, previous TB experience and knowledge of the national tuberculosis guidelines (NTG).^5^ Healthcare providers’ non-compliance with the NTG could contribute to discontent amongst patients and healthcare providers, misuse of scarce resources, a waste of time and missed opportunities for the early diagnosis of TB.^6^

A study conducted in Thailand revealed that 30% of TB patients were not diagnosed according to that country's NTG.^6, 7^ Non-adherence to the NTG included: (1) missing sputum smear examinations (6.8%); (2) TB diagnoses based on three sputum smear-negative results and chest x-ray abnormalities but without any antibiotic trials (12.5%); and (3) TB diagnoses based on fewer than three negative sputum smear results and chest x-ray findings (8.6%). Failure to adhere to NTG during the diagnosis and treatment of TB will increase the TB burden since there will be a higher risk of TB transmission, deterioration of TB patients and more cases of multi-drug resistant TB (MDR-TB).^7^

As many as 97.0% of the smear-negative PTB patients had diagnoses that did not comply with the NTG diagnostic procedures.^8^ This author also reported that of these patients, 24.2% in one region of Ethiopia and 43.4% in another were treated as smear-negative PTB patients, without acid-fast bacillus (AFB) sputum microscopic examinations and without antibiotic trials.

Reportedly, 42.8% of TB diagnoses at Ethiopian hospitals and 60.8% of those at health centres were based on the NTG diagnostic criteria.^8^ Complicated TB cases should be referred to hospitals since hospitals have better resources than clinics. If healthcare providers at hospitals fail to comply with the NTG, the patients’ clinical condition might deteriorate, with the resultant increased risk of spreading TB and MDR-TB throughout Ethiopia.^7^

The recommended weight band classifications for the treatment of TB patients in Ethiopia are: 20 kg – 29 kg, 30 kg – 39 kg, 40 kg – 54 kg, 55 kg – 70 kg and ≥ 70 kg; and anti-TB drugs for all TB patients should be prescribed according to this classification.^9^ In Malawi, a study revealed that 7% of TB patients were prescribed the incorrect number of pills in their anti-TB regimen.^10^

### Problem statement

The Ethiopian Ministry of Health^2^ reported that the national TB case detection rate in 2008 was only 33.9%. This was lower than the WHO's^1^ global target of 70%. Ethiopia's 2008 case detection and treatment success rates were 33.9% and 84%, respectively.^2^ This indicates the necessity of a prompt assessment of healthcare providers’ implementation of the NTG, which could increase TB case detection rates, enhance timely and effective anti-TB treatment and reduce the prevalence of TB and MDR-TB in Ethiopia. Not only would the incidence and prevalence of TB in Ethiopia be decreased, but it would also reduce healthcare expenditure in this resource-limited country.

### Aim of the study

The aim of this study was to assess implementation of the NTG by healthcare providers during diagnosis and treatment of TB patients; to recommend ways to improve TB treatment outcomes; and to reduce the number of patients suffering from TB and MDR-TB.

## Research methods and design

### Approach and design

A descriptive, cross-sectional, retrospective diagnostic audit of TB patients’ medical records was undertaken in four public health facilities, namely, two hospitals and two health centres in Addis Ababa, the capital city of Ethiopia.

### Study site

At the time of the data collection phase of this study, Addis Ababa had five public hospitals and 26 health centres, of which all 26 health centres and two of the regional hospitals were treating TB patients on the DOTS programme. The two participating health centres were selected randomly, whereas the two hospitals were selected purposively, as only those two regional hospitals provided DOTS services.

### Study population and sample

The study population comprised newly-diagnosed TB patients who were at least 15 years old and who had commenced anti-TB treatment at the selected hospitals and clinics from March to November 2010. TB patients who had been diagnosed at other facilities were excluded because the procedures followed during diagnosis were unavailable. Known HIV-positive TB patients were excluded from the study since all HIV-positive persons are screened routinely for TB.

The sample size of 233 was calculated using a simple size determination for single proportion, using the following equation:

$$n=\text{P}\left(1-\text{P}\right)z^{2}/d^{2}$$

where,

*n* = sample size

P = the reliability coefficient 95%

*d* = 0.05 for the precision (based on providers’ reported 45% adherence to the NTG during diagnosis and treatment in Tigray, Ethiopia^10^)

*z* = confidence level or *z*-score of 1.96

These calculations indicated that a total of 212 patients’ records needed to be audited. As some patients’ medical records were expected to be unavailable, a 10% contingency was added, resulting in a final sample size of 233.

Proportionate sampling was used to allocate the records amongst the four participating public health facilities, based on the number of TB patients’ records that met the inclusion criteria. The medical records that met all eligibility criteria were selected randomly, using a table of random numbers. Data were collected using a checklist based on the Ethiopian NTG. The checklist was pre-tested on 10 TB medical records that were excluded from the actual study. No problems were encountered during the pilot testing.

### Data analysis

Data were transcribed anonymously from each TB patient's medical record, using the specifically-designed checklist. Transcribed information included symptoms suggesting TB; physical examinations and findings; laboratory and x-ray tests and their results; treatment with broad-spectrum antibiotics; age; sex; weight; and the types and dosages of drugs prescribed. Based on the documented clinical, physical and laboratory evidence, the diagnosis of each patient was compared with the diagnostic criteria specified by Ethiopia's NTG.

### Ethical considerations

Permission to conduct the study was granted by the Research and Ethics Committee of the Department of Health Studies of the University of South Africa (certificate number 4205-680-2), the Addis Ababa Regional Health Bureau and each participating hospital or clinic, before data collection commenced.

## Results

Of the 233 TB patients, 52.8% (*n* = 123) were men and 47.2% (*n* = 110) were women. Presenting complaints were recorded in 91% (*n* = 212), but the TB diagnoses had only been recorded in 9% (*n* = 21) of these patients’ medical files. Coughs (71.2%; *n* = 151), chest pains (30.2%; *n* = 64), night sweats (29.2%; *n* = 62), fever (27.4%; *n* = 58) and loss of appetite (23.1%; *n* = 49) were the patients’ most frequent reasons for visiting a healthcare facility, according to the other 212 patients’ medical files.

The physical examination findings of only 53.2% (*n* = 124) of the patients had been recorded, implying that nothing had been recorded about the findings from the physical examination findings in the other patients’ files (46.8%; *n* = 109).

The duration of illness was recorded in 80.8% (*n* = 122) of the files of the 151 patients who complained of coughing, showing that 68.9% (*n* = 104) had coughed for more than two weeks. Sputum smear examinations were done for 80.8% (*n* = 84) of these 104 patients, with 48.8% (*n* = 41) being sputum smear positive on two or more slides.

Healthcare providers correctly diagnosed 56.1% (*n* = 69) of the male and 60.9% (*n* = 67) of the female TB patients, according to the NTG. Of the 233 patients, 65.2% (*n* = 152) were diagnosed in hospitals and 34.8% (*n* = 81) at health centres. The diagnostic procedures followed the NTG criteria in 51.3% (*n* = 78) of the hospitals’ patients. The percentage of correctly-diagnosed TB patients, complying with the NTG diagnostic criteria in hospitals, were, by type of TB: smear-positive PTB (93.3%; *n* = 14); smear-negative PTB (4.0%; *n* = 2); and extra-pulmonary TB (EPTB) (71.3%; *n* = 62). Similarly, in health centres, 97.5% (*n* = 39) of smear-positive PTB; 0% (*n* = 0) of smear-negative PTB and 60% (*n* = 9) of EPTB patients were diagnosed correctly in terms of the NTG diagnostic criteria (see Table 1). Of the smear-negative PTB patients, 25% (*n* = 19) were diagnosed using only radiological (x-ray) evidence.

**TABLE 1a:** Correctly-diagnosed new tuberculosis patients by type of health facility (*n* = 233).

Type of TB	Number of records reviewed		

	Hospital	Health centre	Total
Smear-positive PTB	15	40	55
Smear-negative PTB	50	26	76
EPTB	87	15	102

**Total**	**152**	**81**	**233**

TB, tuberculosis; PTB, pulmonary TB; EPTB, extra-pulmonary TB.

**TABLE 1b:** Correctly-diagnosed new tuberculosis patients by type of health facility (*n* = 233).

Type of TB	Diagnosed correctly					

	Hospital		Health centre		Total	
		
	*f*	%	*f*	%	*f*	%
Smear-positive PTB	14	93.3	39	97.5	53	96.4
Smear-negative PTB	2	4	0	0	2	2.6
EPTB	62	71.3	9	60	71	69.6

**Total**	**78**	**51.3**	**48**	**59.3**	**126**	**54.1**

*f*, frequency; TB, tuberculosis; PTB, pulmonary TB; EPTB, extra-pulmonary TB.

Of the 152 TB patients treated at hospitals, 88.2% (*n* = 134) had been prescribed the correct number of anti-TB pills. At health centres, 96.3% (*n* = 78) of the 81 patients had been prescribed the correct number of pills during the initial treatment, as is shown in Table 2.

**TABLE 2:** Prescription of initial anti-tuberculosis drugs by type of facility (*n* = 233).

Type of facility	TB patients prescribed incorrect numbers of anti-TB pills			Number and% of TB patients prescribed the correct number of anti-TB pills		Total	
		
	Dose too high	Dose too low	Total	*f*	%	*f*	%
Health centre	2	1	3	78	96.3	81	34.8
Hospital	4	14	18	134	88.2	152	65.2

**Total**	**6**	**15**	**21**	**212**	**91.0**	**233**	**100.0**

*f*, frequency; TB, tuberculosis.

Of the 21 TB patients who had been prescribed the incorrect number of anti-TB pills, 85.7% (*n* = 18) were from hospitals and of these, 77.8% (*n* = 14) had been prescribed doses lower than the NTG's recommendations. In health centres, 14.3% (*n* = 3) of the patients had been prescribed the incorrect number of pills, with 66.7% (*n* = 2) of these being prescribed anti-TB pills exceeding the NTG's recommended dosages.

The correct numbers of anti-TB pills, in compliance with the NTG treatment recommendations, were prescribed for 91.8% (*n*=101) of the 110 female TB patients and for 90.2% (*n*=111) of the 123 male patients. Of the 233 TB patients, 9% (*n* = 21) were prescribed the incorrect number of pills and of these, 71.4% (*n* = 15) were prescribed anti-TB pills below the recommended dose. The NTG recommends the prescription of anti-TB pills based on the TB patient's body weight and the guideline recommends one and a half, two, three, four or five anti-TB pills for a body weight of 20–29, 30–39, 40–54, 55–70 and over 70 kg, respectively.^1^

Mistakes with regard to the prescription of anti-TB medication were frequent in those TB patients whose pre-treatment weight band ranged from 55 kg to 70 kg – 22.6% (*n* = 14) of these 62 patients had been prescribed incorrect numbers of anti-TB pills. Of the 21 TB patients who had been prescribed incorrect anti-TB medication, 28.6% (*n* = 6) were over-prescribed, enhancing the possibilities of potential drug toxicities; and 71.4% (*n* = 15) were under-prescribed, which could have resulted in treatment failure.

## Discussion

In 9% (*n* = 21) of the TB patients in the study, only their diagnosis had been recorded in the files. Such a dearth of information could impact negatively on communication amongst healthcare providers with regard to patients’ continued care, treatment and follow-up examinations. This lack of information makes it impossible to compare TB patients’ symptoms during and after treatment with their baseline symptoms. In addition, some records failed to portray the duration of the patients’ complaints. The WHO^11, 12^ recommended 100% recording of patients’ presenting complaints and physical examination findings in order to diagnose TB patients correctly. Failure to record patients’ presenting complaints, the duration of illness and physical examination findings might affect the TB patient's treatment and prognosis.

Out of the 212 TB patients whose presenting complaints had been recorded, coughing was the most frequently occurring symptom (71.2%; *n* = 151), which finding was similar to the results of other studies.^13, 14, 15^

Bacteriological examination is the gold standard for TB diagnosis.^16, 17^ Ethiopia's NTG recommends that sputum smear examinations should be repeated in the following situations: (1) three sputum smear examinations were negative; or (2) only one sputum smear was positive before the diagnosis of PTB.^9^ However, in this study, 3.6% (*n* = 2) of the TB patients were diagnosed with smear-positive PTB based on only one sputum smear-positive result, indicating non-adherence with the NTG and resulting in the potential for wrong diagnoses, treatments and the uncontrolled spread of TB.

Of the smear-negative PTB patients, 25% (*n* = 19) were diagnosed using only radiological evidence. This percentage was higher than the 6.9% reported in the Tigray region, but lower than the 41.4% reported in the Amhara region.^4, 8, 10^ In 39.5% (*n* = 30) of the 76 smear-negative PTB patients, no sputum smear examinations were done. This might have resulted in wrong diagnoses of PTB because of the low specificity of clinical signs and radiological findings.^7^

**TABLE 3:** Prescription of initial anti-tuberculosis pills by weight band (*n* = 233).

Weight band in kg	Number of TB patients prescribed incorrect numbers of anti-TB pills			Number and% of TB patients prescribed correct numbers of anti-TB pills		Total	
		
	Dose too high	Dose too low	Total	*f*	%	*f*	%
20–29	0	0	0	0	0.0	0	0.0
30–39	4	0	4	14	77.8	18	7.7
40–54	2	0	2	150	98.7	152	65.2
55–70	0	14	14	48	77.4	62	26.6
Over 70	0	1	1	0	0.0	1	0.4

**Total**	**6**	**15**	**21**	**212**	**91.0**	**233**	**100.0**

*f*, frequency; TB, tuberculosis.

There were no significant variations in the healthcare providers’ level of implementation of the NTG during the diagnosis of TB patients in hospitals or clinics. There was also no major difference in implementation of the NTG treatment recommendations during the initial treatment of TB patients, irrespective of the TB patients’ gender.

Implementation of the NTG initial anti-TB prescriptions of 91% (*n* = 212) was better than the 57.1% reported by a study in Thailand,^10^ but lower than the 93% reported by a study in Malawi.^18^

Fewer healthcare providers at hospitals implemented the NTG during the initial prescribing of anti-TB medication than those at health centres. This could increase the risk of MDR-TB because of the inadequate administration of anti-TB drugs.^7^ Both over- and under-prescription of TB drugs occurred which could lead to treatment failure, the spread of TB throughout communities and the development of MDR-TB. Over-prescription might aggravate the potential side effects of anti-TB drugs, especially hepatotoxicity, which could influence patients to discontinue their treatment, whilst under-prescription would not cure TB and could increase the number of MDR-TB sufferers in the specific communities and in Ethiopia in general.

Healthcare providers who do not implement the NTG treatment recommendations could cause increased numbers of MDR-TB cases in the country, which would have serious healthcare and budgetary consequences. MDR-TB patients require more sophisticated diagnostic and treatment facilities, posing affordability challenges in developing countries.^9^ TB patients whose anti-TB drug dosages exceeded the NTG's recommendations, might encounter severe side effects, increasing the patients’ risk of discontinuing their treatment and increasing the risk of MDR-TB.^19^ Effective implementation of the NTG treatment recommendations could minimise the chances of TB patients progressing to MDR-TB.^1^

## Conclusion

Healthcare providers’ implementation levels of the NTG were low, as only 54.1% (*n* = 126) of diagnoses followed the NTG which could, in turn, contribute to the over-diagnosis of smear-negative PTB and EPTB. Although healthcare providers prescribed the correct regimens and types of anti-TB drugs, 9% (*n* = 21) of the 233 TB patients were prescribed an incorrect number of pills. Under-prescription could result in the increased transmission of TB and MDR-TB, whilst over-prescription could aggravate the drugs’ side effects and cause patients to discontinue their treatment, potentially contributing to the number of MDR-TB cases in Ethiopia.

The healthcare providers’ implementation of the NTG guidelines should be enhanced and sustained in order to prevent TB patients from developing and spreading MDR-TB. Reasons why healthcare professionals fail to implement the NTG should be investigated and addressed. For example, if laboratory or radiographic resources pose challenges to such implementation, these should be discussed with the local and regional healthcare authorities. The NTG might need to be adapted to make its implementation feasible within some resource-limited facilities, specifying referral guidelines to facilities with better resources.

A checklist should be included in every TB patient's file. This could save recording time as the healthcare provider would merely need to indicate the date and result of any test. The numbers and types of anti-TB pills to be prescribed for each weight band should also be indicated on this checklist. Then the healthcare provider would only need to identify the correct treatment (in terms of the patient's weight) and sign in the appropriate place. This could save much time in prescribing the correct treatment for uncomplicated PTB cases. The completion of the checklist, instead of writing paragraphs, could also facilitate and standardise future audits of patient records.

All patients’ TB treatment outcomes should be audited on a regular basis. Identified shortcomings should be addressed during in-service education sessions. Regular audits of TB patients’ medical records could contribute toward enhancing the healthcare professionals’ implementation of the NTG. In addition, they could enhance the effectiveness of TB diagnosis and treatment in Ethiopia. This should contribute to early effective TB diagnoses and treatment, limiting the spread of TB in communities, enhancing the TB treatment outcomes, reducing the number of MDR-TB cases in Ethiopia and reducing healthcare costs.

## References

[1] World Health Organization Guideline for the programmatic management of drug-resistant tuberculosis. Emergency update [document on the Internet]. c2008 [cited 2009 Sep 05]. Available from: http://whqlibdoc.who.int/publications/2008/9789241547581_eng.pdf

[2] Chaisson RE, Martinson NA. Tuberculosis in Africa – combating an HIV- driven crisis. NEJM. 2008;358:1089–1092. http://dx.doi.org/10.1056/NEJMp0800809 18337598 10.1056/NEJMp0800809

[3] Abraha MW, Nigatu TH. Modeling trends of health and health related indicators in Ethiopia (1995–2008): a time-series study. Health Res Policy Syst. 2009;7:29 http://dx.doi.org/10.1186/1478-4505-7-29 20003381 10.1186/1478-4505-7-29 PMC2797494

[4] Mengiste M, Tesfay T, Madeley R. The quality of tuberculosis diagnosis in districts of Tigray region of northern Ethiopia. Ethiop J Health Dev. 2005;7:13–20.

[5] Shimeles E, Aseffa A, Yamuah H., et al Knowledge and practice of private practitioners in TB control in Addis Ababa, Ethiopia. Int J Tuberc Lung Dis. 2006;10(10):1172–1177. 17044213

[6] Marquez L. Helping healthcare providers perform according to standards [document on the Internet]. c2001 [cited 2014 Jun 06]. Available from: http://pdf.usaid.gov/pdf_docs/PNACN246.pdf

[7] World Health Organization Diagnostic and treatment delay in tuberculosis: an in-depth analysis of the health-seeking behaviour of patients and health system response in seven countries of the Eastern Mediterranean Region [document on the Internet]. c2006 [cited 2009 Sep 05]. Available from: http://www.emro.who.int/dsaf/dsa710.pdf

[8] Birara T. Process evaluation of quality of care in the diagnosis and treatment of TB in Noth Wollo Zone of the Amhara region, Northern Ethiopia. Unpublished master’s dissertation. Faculty of Science, Department of Health Studies. Jimma: Jimma University: 2007.

[9] Ministry of Health (Ethiopia) Guideline for program and clinical management of drug resistant tuberculosis [document on the Internet]. c2009 [cited 2014 Jun 14]. Available from: www.etharc.org/resources/downloads/finish/66/367

[10] Harries AD, Gausi F, Salaniponi FM. Prescriptions and dosages of anti-tuberculosis drugs in the National Tuberculosis Control Programme of Malawi. Int J Tuberc Lung Dis. 2004;8(6):724–729. 15182142

[11] World Health Organization Brief guide on tuberculosis control for primary health care providers [document on the Internet]. c2004 [cited 2009 Oct 24]. Available from: http://www.euro.who.int/document/e82858.pdf

[12] World Health Organization Pathway to better diagnostics for tuberculosis: a blueprint for the development of TB diagnostics [document on the Internet]. c2009 [cited 2010 Mar 05]. Available from: http://www.stoptb.org/wg/new_diagnostics/assets/documents/BluePrintTB_annex_web.pdf

[13] Cambanis A, Ramsay A, Wirkom V., et al Investing time in microscopy: an opportunity to optimise smear-based case detection of tuberculosis. Int J Tuberc Lung Dis. 2007;11(1):40–45. 17217128

[14] Chung WS, Chang RE, Guo HR. Variations of care quality for infectious pulmonary tuberculosis in Taiwan: a population based cohort study. BMC Public Health. 2007;7:107 http://dx.doi.org/10.1186/1471-2458-7-107 17562022 10.1186/1471-2458-7-107 PMC1906756

[15] Farah MG, Rygh JH, Steen TW., et al Patient and health care system delays in the start of tuberculosis treatment in Norway. BMC Infect Dis. 2006;6:33 http://dx.doi.org/10.1186/1471-2334-6-33 16504113 10.1186/1471-2334-6-33 PMC1435913

[16] Canan ED, Tanrikulu AC, Acemoglu H., et al A multicentre study of doctors’ approaches to the diagnosis and treatment of tuberculosis in Turkey. J Infect Dev Ctries. 2009;3(5):357–364. 19759505 10.3855/jidc.243

[17] World Health Organization International standards for tuberculosis care [document on the Internet]. c2006 [cited 2009 Nov 21]. Available from: http://www.who.int/management/quality/standards/InternationalStandardsTBCare.pdf

[18] Thongraung W, Chongsuvivatwong V, Pungrassamee P. Multilevel factors affecting tuberculosis diagnosis and initial treatment. J Eval Clin Pract. 2008;14(3):378–384. http://dx.doi.org/10.1111/j.1365-2753.2007.00871.x 18373583 10.1111/j.1365-2753.2007.00871.x

[19] Jittimanee SX, Madigan EA, Jittimanee S., et al Treatment default among urban tuberculosis patients, Thailand. Int J Nurs Pract. 2007;13(6):354–362. http://dx.doi.org/10.1111/j.1440-172X.2007.00650.x 18021164 10.1111/j.1440-172X.2007.00650.x

